# Supplementary material for: SARS-CoV-2 Nucleocapsid Protein Is Not Responsible for Over-Activation of Complement Lectin Pathway
Source: Int J Mol Sci. 2024 Jul 4;25(13):7343. doi: 10.3390/ijms25137343 (PMC11242754; doi:10.3390/ijms25137343)
Supplement: Supplementary file 1 [file ijms-25-07343-s001.zip › ijms-3043393-supplementary.pdf]

Supplementary Materials

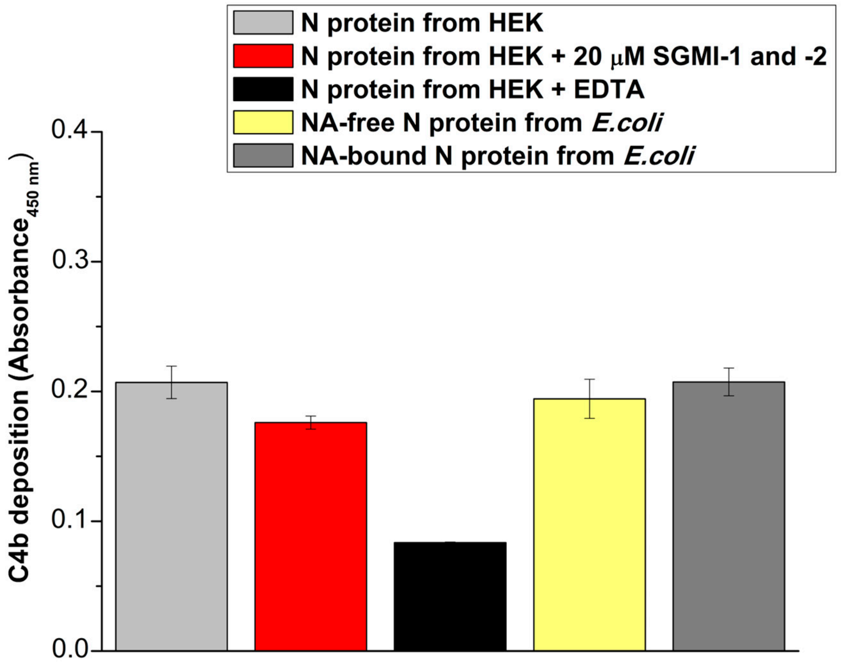

Figure S1. Complement activation on N protein expressed in mammalian cells. Microtiter plates were coated with N protein expressed in HEK cells or *E. coli* cells. C4b deposition was initiated in 10% NHS. LP-specific inhibitors, SGMI-1 and -2, had no effect on the complement activation. EDTA inhibits LP and CP activity therefore NHS-EDTA served as negative control.

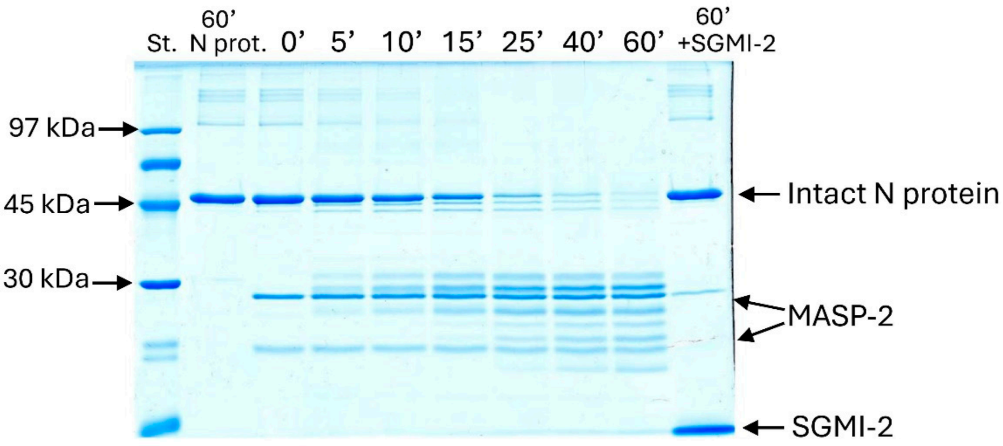

Figure S2. The cleavage of N protein by MASP-2. 2.8 μM N protein was incubated with 0.75 μM MASP-2 at 37 °C, samples from the reaction were taken at different timepoints and run on SDS-PAGE under reducing conditions. The amount of intact N protein decreases over time while degradation products appeared. MASP-2 inhibitor SGMI-2 prevented proteolytic cleavage.

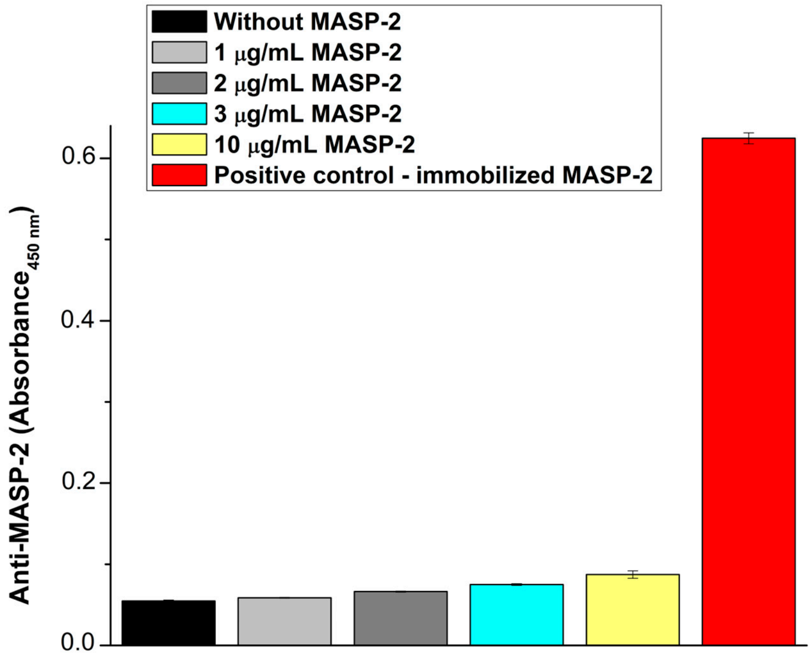

Figure S3. MASP-2 binding to immobilized N protein. Microtiter plates were coated with 10 μg/mL N protein. Wild type MASP-2 (CCP1-CCP2-SP) was added to wells at different concentrations: 1 μg/mL (light gray); 2 μg/mL (gray); 3 μg/mL (cyan); and 10 μg/mL (yellow). Wells were coated with N protein and incubated with buffer instead of MASP-2 served as negative control (black), while immobilized MASP-2 represented positive control for anti-MASP-2 antibody (red). Experiments were repeated twice; error bars show standard deviation of triplicates in a single measurement.
